# Phloretin in Benign Prostate Hyperplasia and Prostate Cancer: A Contemporary Systematic Review

**DOI:** 10.3390/life12071029

**Published:** 2022-07-11

**Authors:** Che-Hsueh Yang, Yen-Chuan Ou, Chi-Chien Lin, Yi-Sheng Lin, Min-Che Tung, Chia-Cheng Yu, Jen-Tai Lin, Chen-Yueh Wen

**Affiliations:** 1 Division of Urology, Department of Surgery, Tungs’ Taichung MetroHarbor Hospital, Taichung 435, Taiwan; b101098093@tmu.edu.tw (C.-H.Y.); ycou228@gmail.com (Y.-C.O.); t12197@ms.sltung.com.tw (Y.-S.L.); t1142@ms.sltung.com.tw (M.-C.T.); 2 Institute of Biomedical Science, The iEGG and Animal Biotechnology Center, National Chung-Hsing University, Taichung 402, Taiwan; lincc@email.nchu.edu.tw; 3 Division of Urology, Department of Surgery, Kaohsiung Veterans General Hospital, Kaohsiung 813, Taiwan; mlee0857@gmail.com

**Keywords:** phloretin/pharmacology, phloretin/therapeutic use, prostatic neoplasms/drug therapy, prostatic hyperplasia/drug therapy, male

## Abstract

Currently, medication for benign prostate hyperplasia (BPH) and prostate cancer (PCa) are mainly based on modulating the hormone and nervous systems. However, side effects often affect patients, and might decrease their commitment to continuing the medication and lower their quality of life. Some studies have indicated that chronic inflammation might be the cause of BPH and PCa. Based on this hypothesis, the effect of phloretin, a potent anti-inflammatory and anti-oxidative flavonoid, has been researched since 2010. Results from animal and in-vitro studies, obtained from databases, also indicate that the use of phloretin in treating BPH and PCa is promising. Due to its effect on inflammatory cytokines, apoptosis or anti-apoptosis, reactive oxygen species, anti-oxidant enzymes and oxidative stress, phloretin is worthy of further study in human clinical trials regarding safety and effective dosages.

## 1. Introduction

Phloretin, a flavonoid extracted from apple leaves, has been widely researched for its anti-inflammatory and anti-tumoral effects in many preclinical studies [1,2]. The biochemical mechanism of antioxidant in phloretin has been explored from various aspects, and its treatment effects were determined by its O-H bonds. Based on density functional theory (DFT), there are two prime indicators in assessing antioxidant capacity: bond dissociation enthalpy (BDE) and ionization potentials (IPs). BDE represents the energy needed to break a bond into two isolated atoms or molecules and is part of the endothermic process. It is easier to destruct the bonds in low BDE than in high BDE. Thus, low BDE could represent the better anti-oxidant capacity. On the other hand, IPs indicate the energy needed to deprive an electron of its quiescent atoms or molecules and represents the efficiency of the singlet-oxygen quenching and scavenging of free radicals.

**Figure 1:**
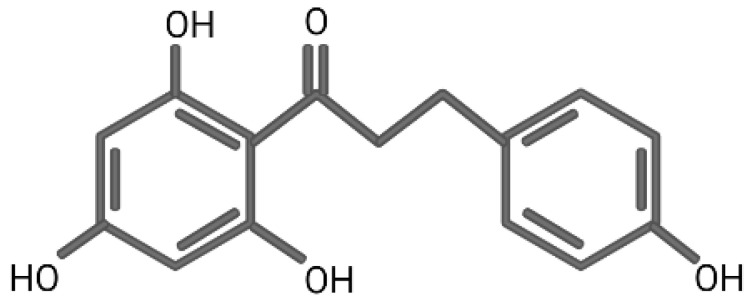
The chemical structure of phloretin.

In DFT, hybrid functionals are classes of approximation to the exchange–correlation energy functional. The exact theories applied are named after the functionals used in one exchange-correlation functional. For example, in B3LYP, B represents the exchange functional of Becke, and 3 represents three parameters. LYP represents the correlation functional of Lee-Yang-Parr. Since the alteration of the exchange functional of Becke, the approximation of B3LYP would be more precise than that of BLYP. Accompanying the theory, a basis set is adopted to calculate the energy. This way, the total methodology applied in estimating the energy of the bonds of certain molecules would be addressed as the theory/basis set. In phloretin, there are four O-H bonds (Figure 1) in its molecular structure. In water, according to the theory of B3LYP and the basis set of 6-311 + G(d,p), the smallest BDE of O-H bond was 79.36 kcal/mol, while the BDE level of gallic acid in water was 90.93 kcal/mol. Since the BDE of gallic acid was deemed as the anti-oxidant reference in the experimental examination, this result demonstrated that phloretin could possibly possess an outstanding anti-oxidant capacity [3]. When comparing the solvents, a study demonstrated that a polar environment would be better than a non-polar, and water would be the optimal solvent, providing the least IPs, at 139.48 kcal/mol, among all tested solvents with the B3LYP/6-311 + G(d,p) basis set. In summary, judging from the BDE and IPs, there are few energy costs to subtracting an electron from phloretin in water for the scavenging of free radicals and quenching of a singlet oxygen, indicating its remarkable anti-oxidant role [3]. In a study of the in-vitro human cancer cell line, it could prevent oxidative DNA damage and induce anti-oxidant enzyme expression [4].

In anti-proliferative assessment, the pro-apoptotic effect of phloretin was induced by increasing the expression of Bcl-2 associated X protein (Bax), which subsequently downregulated the mitochondrial membrane potential and upregulated the level of caspase-3 and -9 [5]. In cell lines of non-small cell lung cancer, it was reported that phloretin could suppress the level of Bcl-2, and provide an additional positive effect along with cisplatin on inhibiting the proliferation of cancer cells [6]. Nowadays, more and more evidence has indicated that a local or systemic immune response was related to cancerous development or progression [7]. Among all transcription factors, the kappa-light-chain-enhancer of activated B cells (NF-κB) was found to be the common key role in cancer cells and immune cells, and could trans-activate the embedding protein involved in tissue inflammation and oncogenesis [8]. Based on this, it appears that phloretin could further exert its comprehensive role in anti-inflammatory and anti-tumoral responses by inhibiting the activation of NF-κB [9,10]. The general introduction of phloretin is summarized in Table 1. In this article, we provide the first comprehensive review of the role of phloretin in prostate cancer (PCa) and benign prostate hyperplasia (BPH), and elaborate our viewpoint of its future clinical application. 

**Figure 2:**
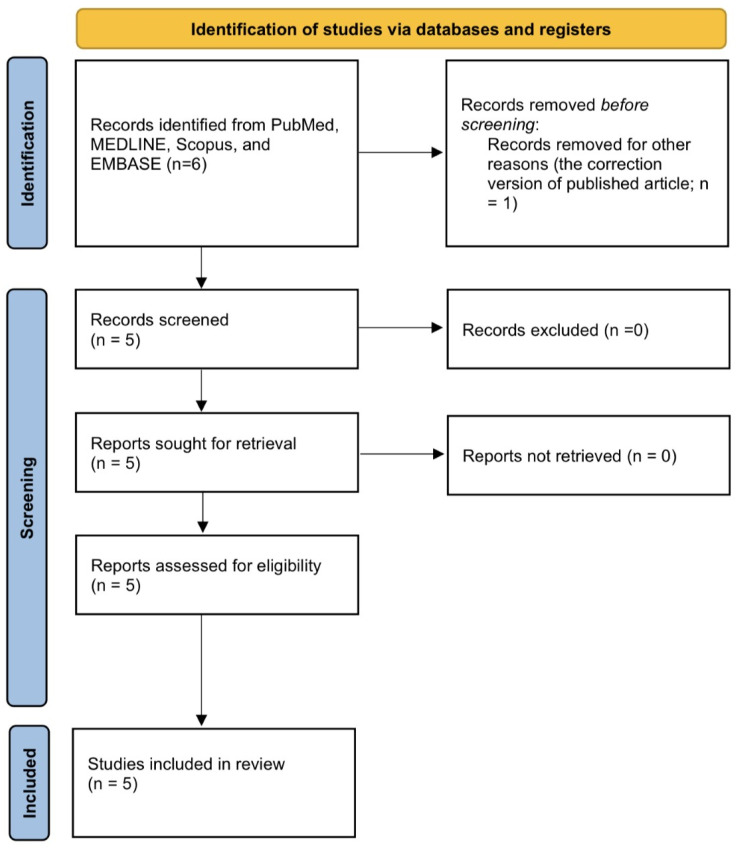
PRISMA flow diagram for searching and screening of the articles.

To search the related articles on this topic, we used the search term “(phloretin) AND ((benign prostate hyperplasia) OR (prostate cancer))” with all fields in PubMed, MEDLINE, and EMBASE (Figure 2). There are a total of six results with five original articles available [11,12,13,14,15], and the full texts of these five articles are available.

## 2. Phloretin, BPH, and PCa

**Figure 3:**
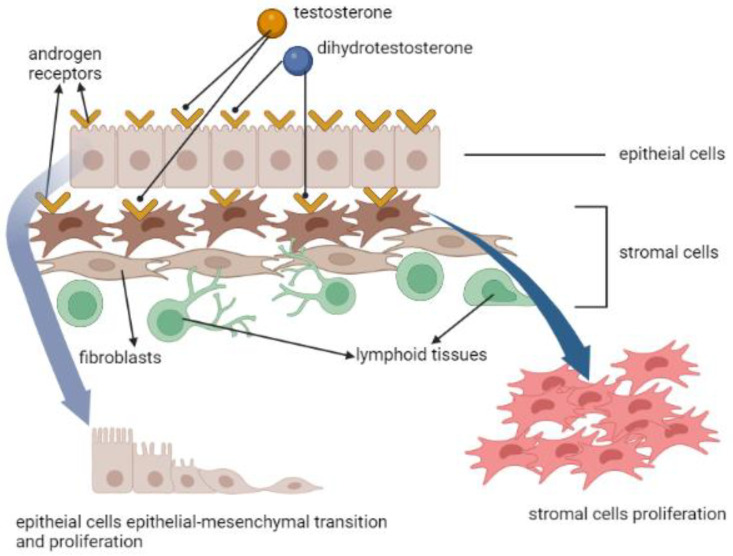
The effects of androgen receptor, testosterone and dihydrotestosterone in prostate cells’ proliferation and epithelial-mesenchymal transition.

In the development of normal prostate [16], BPH [17], and PCa [18], the influence of androgen receptor (AR) is most commonly mentioned. The prostate consists of the stromal cells and epithelial cells, and the former include fibroblasts, smooth muscle cells and some inflammatory cells, such as human prostate-associated lymphoid tissue. AR exists in both epithelial cells and stromal cells. After testosterone (T), or its metabolite dihydrotestosterone (DHT) by 5α-reductase in stromal cells, binds to the AR in cytoplasm, it will enter the nucleus, causing a signal cascade afterwards [19]. Aside from cellular growth and proliferation, one of the most essential changes is the epithelial-mesenchymal transition (EMT), making the cellular adhesion loose and allowing cellular migration [20] (Figure 3). This pathophysiology could be related to BPH [21], and the worst scenario is to cause tumor stemness and aggressiveness [22]. 

### 2.1. Immune Microenvironment in BPH

Infiltrating macrophages were hypothesized as enhancing stromal growth and leading to BPH via AR signaling [23]. Moreover, this effect was more significant in the transitional zone [24]. In animal model, chemokine (C-C motif) ligand 3 (CCL3), also known as macrophage inflammatory protein 1-α (MIP-1α), was observed to be essential not only in the macrophages’ infiltration and migration, but also crucial in inducing stromal cells’ proliferation. Meanwhile, it was also noted that CCL3 in human prostate tissue of BPH was higher than that of non-BPH, suggesting the potential effect of CCL3 in macrophage-mediated stromal cells’ proliferation [23]. 

**Figure 4:**
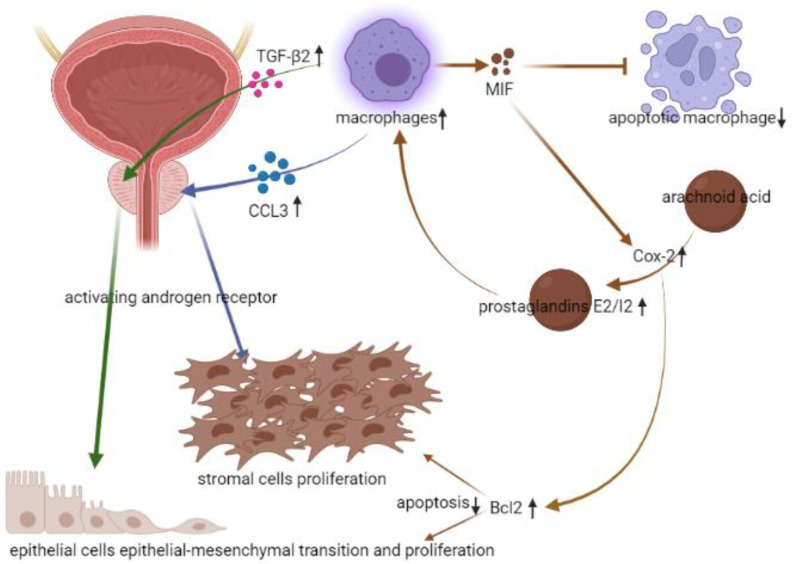
The relation between infiltrating macrophages and BPH. (↓: decreased, ↑: increased).

Asides from the stromal cells, infiltrating macrophages were also noted as existing in epithelial cells, and AR-triggered EMT in epithelial cells could be activated by transforming growth factor β2 (TGF-β2) [25]. This interaction between macrophages and AR in prostate epithelial cells could also contribute to BPH. To sustain the pro-inflammatory microenvironment, activated macrophages would give off macrophage migration inhibitory factor (MIF), which further suppressed the p53-dependent apoptosis, induced the arachidonic acid metabolism, and produced cyclooxygenase-2 (Cox-2) [26]. The anti-apoptotic effect of MIF and the subsequent elevated prostaglandins E_2_/I_2_ would make macrophages exist for longer. The sustained macrophages would keep stimulating BPH via AR signaling. Another effect of stimulated Cox-2 would be related to the upregulation of Bc1-2, which would further cause proliferative and anti-apoptotic ability of prostate epithelial cells [27] (Figure 4.). Thus, there was also a study proposing that inhibition of MIF might be another potential strategy in treating BPH [28].

### 2.2. Immune Microenvironment in PCa

In PCa, the role of androgen is crucial from the initial assessment, androgen deprivation therapy (ADT), to survival prediction. In a previous published study [29], it was found that some motifs, such as Forkhead Box A1 (FOXA1) and Homeobox B13 (HOXB13), in AR malignant shift were more abundant in PCa than the normal prostate tissue, and this shift would further lead to the transcription of cancerous phenotype. Aside from this, AR amplification could be more commonly seen in recurrent PCa [30], ADT-treated PCa [31], metastatic PCa [32] and castration-resistant PCa [33]. Interestingly, not all occasions of AR amplification are associated with the poor survival outcome [30,31]. 

**Figure 5:**
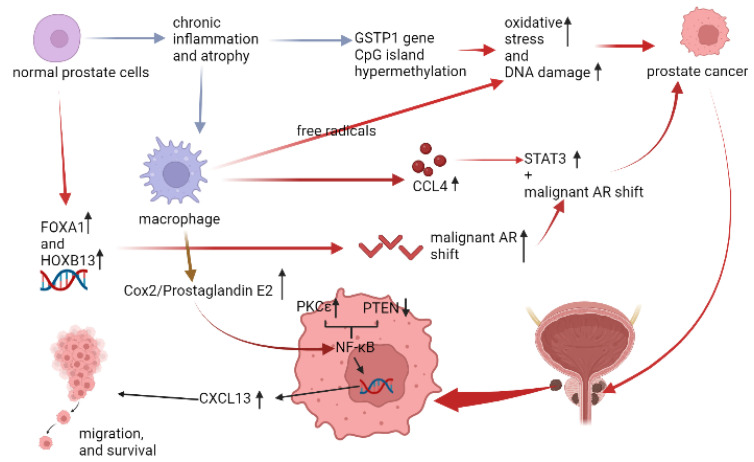
The relation between infiltrating macrophages and PCa. (↓: decreased, ↑: increased).

Although AR malignant shift is related to the tumorigenesis of PCa, the clear interaction still needs to be elucidated [34]. In gross histology, chronic inflammation and atrophy were observed close to the site of PCa [35], and approximately 60% of men with low-grade PCa had both chronic inflammation and prostate atrophy of this feature [36]. Moreover, this feature would be more frequently seen at the peripheral lobe [37]. In the aspect of immune microenvironment, one of the characteristics of prostate atrophy was the hypermethylation of CpG island in glutathione S-transferase-pi (GSTP1) gene. GSTP1 gene CpG island hypermethylation was thought to be the early change in the tumorigenesis of PCa [38], and was barely observed in normal prostate epithelial cells and hyperplastic cells [39]. Normally, the product of GSTP1 gene is glutathione S-transferase, an anti-oxidative enzyme releasing the inflammatory oxidative stress (OS) from reactive oxygen species (ROS). The imbalance between detoxification and ROS would cause DNA damage and initiate tumorigenesis. Besides, infiltrating macrophages in prostate stromal cells could provide additional free radicals to cause the DNA damage [40], and directly initiate tumorigenesis via AR- mediated carbon tetrachloride/signal transducer and activator of transcription 3 (CCL4/STAT3) signaling [41]. In another aspect, overexpressed pro-oncogenic kinase, protein kinase Cε(PKCε), and phosphatase and tensin homolog (PTEN) loss would synergically activate the NF-κB pathway [42], and the NF-κB pathway was related to the migration and survival of PCa [43,44]. Moreover, the abovementioned NF-κB pathway was all mediated by Cox-2 [42,43,44], and this tumorigenesis of Cox-2 might also be effective in androgen-independent PCa [45] (Figure 5). Recently, the study from liquid biopsy of peripheral blood samples of human demonstrated that the level of Cox-2 might be associated with the diagnosis and prognosis of PCa [46]. 

### 2.3. Phloretin and the Current Medical Options for BPH

BPH is one of the most common diseases among middle-aged men. It occurs in 15% to 60% of men aged 40 and older, and the prevalence increases to 70–80% after the age of 80 [47,48]. The obstructive uropathy of BPH has been attributed to both static and dynamic factors. The static obstruction is due to the hyperplastic prostatic tissue compressing the prostatic urethra and bladder outlet, whereas the dynamic obstruction is related to the adrenergic nervous system and prostatic smooth muscle tension [47,49]. As mentioned earlier, androgens in BPH play an important role in the differentiation of prostatic growth [48,50]. At present, therapeutic options for BPH include lifestyle modification, drug therapy and surgical intervention. 

Recently, there have been various medical options for BPH. The primary goal was to achieve either prostate smooth muscle relaxation or shrinkage of enlarged prostate. The most frequently prescribed first-line medical therapies are α-blockers, either selective or non-selective, and 5α-reductase inhibitors [51]. The mechanism of α-blockers is to relax smooth muscle in the prostate stroma, which could improve urinary flow rate. However, it might cause side effects such as orthostatic hypotension, retrograde ejaculation, and dizziness [52]. The therapeutic theory of 5α-reductase inhibitors is to decrease intraprostatic levels of DHT. This inhibitory effect will subsquently lead to the suppression of prostatic growth and apoptosis. However, its side effects, such as disorder ejaculation, erectile dysfunction, and loss of libido will greatly affect the quality of life and lower patients’ compliance [53]. The most commonly discussed medication other than α-blockers and 5α-reductase inhibitors are the phosphodiesterase type 5 inhibitors (PDE5i), such as tadalafil. Since phosphodiesterase type 5 (PDE5) would spread in the penile corpora cavernosa, urinary bladder, urethra, and prostate, its therapeutic mechanism was proposed as an increase in oxygenation, a decrease in prostate inflammation, and relaxation of the smooth muscle [54]. In the clinical assessment, its benefits included improving both symptomatic scores of lower urinary tracts symptoms (LUTS) and erectile function. Moreover, it was well tolerated among participants in clinical trials and few discontinuations due to side effects were reported [55]. BPH, aging, and erectile dysfunction were three conditions that would coexist. Unlike α-blockers and 5α-reductase inhibitors, causing sexual dysfunction, PDE5i has gained attraction in clinical practice for improving BPH with LUTS and erectile dysfunction at the same time.

If patients have repeated urinary retention, post-renal insufficiency, and failure of medical control, surgical intervention would be indicated. Transurethral resection of the prostate (TURP) is currently the gold surgical method for BPH. However, TURP has the risks of perioperative complications such as transfusion, TUR syndrome, blood clot retention and urinary tract infection. Late postoperative complications include stress incontinence, retrograde ejaculation, urethral stricture and bladder neck contracture [56].

Currently, some of the literature has found that the development of BPH in the elderly is not only caused by the hormone pathway, but is also closely related to the inflammatory and metabolic microenvironment [49,57]. Metabolic or hormonal abnormalities could further transform the acute inflammation from infectious episodes into chronic inflammation. In reponse to chronic inflammation, the provoked adaptive immunity in prostatic stroma would directly aggregate the infiltrating macrohpages, or induce Th17, Th1 and Th2 lymphocytes to secrete cytokines. For example, Th2 lymphocyte would activate M2 macorphages and induce EMT via the TGF-β pathway, and Th1 would activate M1 macrophages via secreting interferon gamma (IFN-γ). The activated M1 macrophage would then cause the increased ROS directly or indirectly by secreting TNF-α, IL-1, and IL-6. The secreated TNF-α, IL-1, and IL-6 would cause the production of Cox-2, and the increased ROS would further stimulate TGF-β. Besides, Th17 lymphocytes would release IL-17 and IL-8 to attract polymorphonuclear lymphocytes and stimulate NF-κB. The stimulated NF-κB would further enhance the inducible nitric oxide synthase (iNOS) and produce reactive nitrogen species (RNS). Consequently, lipidic peroxidation would occur due to elevated RNS. Aside from Th17, the elevated ROS could also induce the production of RNS via the NF-κB pathway [58]. Based on this pathophysiology, several studies have tried to use phytotherapy in the BPH treatment to improve the immune microenvironment.

In the present study of a rat model with T-induced BPH, a dosage of 100 mg/kg/day for 28 days could ameliorate the hyperplastic condition in gross anatomy [11]. In hormone, DHT was lowered, but the 5α-reductase was not interfered with. In inflammatory cytokines of IL-6, IL-8, Il-17, NF-κB and Cox-2 were all elevated in the BPH group. This implied the underlying inflammatory influence on inducing BPH. As for lipidic peroxidative product, malondialdehyde was significantly elevated. This model classically reflected the current immune microenvironment regarding the chronic inflammatory effect on BPH [27,59]. By administrating phloretin at 100 mg/kg/day, these inflammatory cytokines and pathways were all downregulated, and the antioxidants were elevated to release the OS. In proliferative assessment, anti-apoptotic Bcl-2 was downregulated, and pro-apoptotic Bax and caspase-3 were upregulated [11]. However, when the dosage of 50 mg/kg/day was administered, only partial inhibitory effects on BPH were observed. In gross histology, the thickness of protate epithelial cells were not remarkably decreased. Although IL-6, IL-8, and Il-17 were downregulated, NF-κB and Cox-2 were not lowered. As mentioned above, Cox-2 would be related to the upregulation of anti-apoptotic Bcl-2, and insufficient suppression of Bcl-2 would cause proliferative epithelial cells, reflected in the gross histology of T-induced BPH with phloretin of 50 mg/kg/day [11]. Aside from insufficient suppression of Bcl-2, both NF-κB and Cox-2 were associated with the regulation of oxidative stress (OS), and a non-significant decrease of was also related to the increased OS. In conclusion, by reversing the inflammatory changes, inducing apoptosis, and decreasing OS, 100 mg/kg/day phloretin was effective in controlling T-induced BPH in an animal model.

### 2.4. The Current Medical Options for PCa

PCa is one of the most common cancers in males. It is characterized by an unpredictable outcome in the intermediate and high-risk groups of localized PCa, and high mortality rate in the metastatic PCa. In 2021, it was the second leading cause of male cancer death [60]. As mentioned before, T, DHT and AR are important components in developing PCa [61]. Therapeutic weapons against PCa include surgery, radiation, chemotherapy, immunotherapy, and ADT. According to the latest National Comprehensive Cancer Network guideline, ADT could be used in combination or alone in the unfavorable intermediate and high regional risk group, increasing PSA or lymph node positivity after radical prostatectomy and metastatic PCa. To control PCa, ADT could reduce the circulating level of T by affecting the hypothalamic–pituitary–gonadal axis. Among various choices, Gonadotropin-releasing hormone (GnRH) agonist is the most common ADT, motivating the GnRH receptor and its desensitization, and suppressing the GnRH release. GnRH antagonist is another ADT blocking the GnRH receptor and decreasing GnRH secretion [62,63]. However, the side effects of ADT include osteoporosis, risk of fracture, hot flushes, metabolic syndrome, sexual dysfunction, and cardiovascular events [64]. Additionally, ADT for PCa could possibly lead to AR mutation and make PCa castration-resistant. Recently, the developments of novel AR pathway-directed therapies, such as Abiraterone acetate, Enzalutamide, Apalutamide, and Darolutamide have been approved for castration-resistant PCa. Although new hormone agents have been developed rapidly, several adverse events are reported such as skin rash, fatigue, hypertension, cognitive function, seizure, and even cardiovascular events [65,66,67]. The chemotherapeutic agent, docetaxel, is utilized as another treatment for improving overall survival of metastatic or castration-resistant PCa [68,69]. Its side effects are febrile neutropenia, hypersensitivity reactions, fluid retention, and nail changes. Among these side effects, febrile neutropenia could threaten men with infectious episodes and could be lethal [70]. However, some aggressive PCa conditions might keep progressing even after these drugs are used. For these extreme conditions, more and more remedies have been invented, such as Poly (ADP-ribose) polymerase (PARP) inhibitor Olaparib. In men with castration-resistant PCa and BRCA1, BRCA2, or ATM mutations, Olaparib could be considered when they have disease progression after receiving new hormonal agents or chemotherapies. However, uncertain side effects and expensive cost are two important factors in clinical uses. Even then, new remedies for PCa are still necessary. In the immune microenvironment, the relationship between the human microbiome and PCa has been explored. The microbiome helps modulate the immune system and hormone levels in circulation that might influence cancer risk, response to ADT and progression [71]. Thus, modulating the immune microenvironment might be another useful way to develop medication for PCa.

PC3 and DU145 cell lines are bone metastatic and androgen insensitive PCa. The former has high tumorigenicity and the latter more moderate. 100-μM phloretin was able to suppress cancer cells’ proliferation and migration for both. However, contrary to the inhibitory effect on ROS in a T-induced BPH animal model [11], 100-μM phloretin inhibited the PCa progression through generating ROS [12]. Phloretin would not only increase the ROS in human PCa cell lines, but it could also decrease the effect of the antioxidant detoxification agent, such as superoxide dismutase (SOD), glutathione peroxidase (GPx), and catalase. These regulatory changes were also contrary to that of phloretin in a T-induced BPH animal model [11]. Aside from the ROS, mRNA expressions of Wnt/β-Catenin signaling pathway associated with EMT in PCa were also downregulated [12]. This interesting relation between increasing ROS and decreasing PCa cell growth is obviously contrary to the hypothesis we mentioned previously. It was supposed that this experimental finding might be attributed to the different ROS characteristics in the different PCa stages [72,73,74]. The relation between antioxidants and PCa is complicated. The anti-tumoral effect of antioxidants in prostate cells with normal prostate-specific antigen (PSA) would be reversed into pro-tumoral effect in prostate cells with high PSA [75,76,77], which was the most common for PCa. Another mechanism of treatment effect of phloretin in PC3 cell line was that it could efficiently induce apoptosis [13]. In the concentration of 100 μM, they also found that anti-apoptotic proteins, such as Bcl-2, were decreased and pro-apoptotic proteins, such as Bax and caspase-3, were increased [13].

Asides from the PC3 and DU145 cell lines, LNCaP features PCa without bony metastasis and low tumorigenicity. Contrary to PC3 and DU145 cell lines, it appears to be androgen sensitive. By inducing apoptosis, phloretin could offer its anti-tumoral effect in LNCaP cell lines [13]. Moreover, by inhibiting MEK/ERK1/2 pathways, Cox-2 expression could be downregulated in PCa [13], since the Cox-2 mediated-NF-κB pathway was related to the survival and migration of PCa cells [42,43,44], which was also effective in androgen-independent PCa. This characteristic might empower phloretin to promote an inhibitory effect on androgen-independent PCa.

Aside from exhibiting an anti-tumoral effect via regulating the immune microenvironment, phloretin was also observed as enhancing cytotoxic and apoptotic effects in androgen sensitive PCa cells by mediating the tumor necrosis factor-related apoptosis-inducing ligand (TRAIL) [14]. In cellular metabolic features for survival, glucose transporter 1 (GLUT1) is responsible for glucose uptake in cancer cells by a mechanism of facilitated diffusion. Based on this mechanism, phloretin could inhibit GLUT1 in both androgen sensitive LNCaP and insensitive PC3 cells. It was found that modifying GLUT1 expression in androgen sensitive PC3 cells up to 50% was the most effective among different flavonoids [15]. In conclusion, through different anti-tumoral mechanisms, such as increasing ROS and inhibiting the Cox-2 mediated NF-κB pathway, phloretin might have potential in providing the next medication for AR-dependent or independent PCa. The general inhibitory details of phloretin among different cell lines are summarized in Table 2.

## 3. Perspective of Future Studies of Phloretin in Treating BPH and PCa

Based on the hypothesis that BPH and PCa might possibly originate from chronic prostatitis, the anti-inflammatory ability of phloretin has potential in preventing both BPH and PCa. In past animal and in-vitro studies [11,12,13,14,15], phloretin could ameliorate both of these with similar mechanisms, which were anti-inflammatory, anti-proliferative, and modulating OS. Although the inflammatory response linking BPH to PCa still remains unclear [78], some determinant cytokines, such as Cox-2, were found to be meaningful [79]. For example, over-expressive Cox-2 would subsequently lead to enhanced anti-apoptotic Bcl-2 and angiogenetic VEGF, and then give cells potential tumorigenic features. From the same studies, it was noted that modulating the immune microenvironment seemed to be a feasible solution, aside from the conventional medication, in BPH and PCa, and perhaps would benefit patients, with fewer side effects.

Another possible common mechanism in BPH and PCa is accumulation of senescent cells. In the process of ageing, senescent cells gather in all kinds of tissues, including prostate, and their secretory phenotype would cause a pro-inflammatory microenvironment in prostate. Based on current studies, these senescent cells in prostate are most likely to exist in epithelial cells [80]. Furthermore, the cytokine IL-6 and chemokine IL-8 were possibly secreted by the senescent cells and contributed to both BPH and PCa [80]. In an animal model of T-induced BPH [11], phloretin could remarkably reduce the concentration of IL-6 and IL-8, implicating the possible sero-lytic effects of phloretin in BPH. However, since other pro-inflammatory biomarkers by senescent cells were not examined in the T-induced animal model [11], the sero-lytic effects of phloretin remained unclear in BPH. As for PCa, several transducers created by the senescent cells’ pro-inflammatory microenvironment were proposed to be related to the oncogenic phenotypes [80]. Based on the proposed pathways and studies regarding phloretin in PCa [12,13,14,15,80], the sero-lytic effect of phloretin in PCa was possibly exerted via down-regulating the wnt/β-catenin signaling pathway. However, regardless of BPH or PCa in the current studies, none of the animal models and in-vitro studies were specifically designed to assess the senescent cells in prostate and the sero-lytic effects of phloretin. Herein, although the sero-lytic effects of phloretin in prostate possibly existed, more specific studies regarding this topic are still needed.

From the study of an animal model and in-vitro cells, there were limited clues to detecting an effective dosage in BPH and PCa. In the only published study of this in BPH, a dosage of 100 mg/kg/day would exert its full potential on inhibiting T-induced BPH in rats, and half of that dose would be inadequate. However, whether there is any effective dosage between 50 to 100 mg/kg/day remains unknown [11]. For PCa, there was more information retained from the published studies. 100-μM phloretin could be effective in inhibiting androgen sensitive LNCaP cells, insensitive PC3 cells, and metastatic DU145 cells [12,13,14,15]. In IC_50_, this was 25 μM and 39.4 μM in androgen sensitive LNCaP cells and insensitive PC3 cells, respectively. When androgen sensitive LNCaP cells were induced to be hormone-resistant, as in LNCaP-R, the IC_50_, would be elevated to29.4 μM. When the stemness of insensitive PC3 cells was attenuated [81], as in PC3-AR, the IC_50_ would decrease to 37.1 μM from 39.4 μM [15]. One of the exciting results was that even in the PCa of hormone-resistant status, induced by androgen from originally hormone-sensitive status, phloretin still possessed treatment effectiveness. This allows for the possibilities of it being utilized in castration-resistant PCa in the future.

However, potential side effects were not mentioned in the present studies of phloretin in BPH and PCa. In BPH, as mentioned earlier, the adherence to medication among patients was mainly determined by side effects. With α-blockers, patients were bothered by retrograde ejaculation. As for 5α-reductase inhibitors, erectile dysfunction and loss of libido were reported by patients on the medication. Judging from the immune-modulatory mechanism of phloretin in BPH, retrograde ejaculation would be less than with α-blockers. Sexual function and the potential side effects regarding this would be of great importance when developing drugs for BPH and PCa, since the population suffering from BPH and PCa largely overlapped with the population suffering from erectile dysfunction [82,83]. Although 5α-reductase enzyme was not lowered by phloretin, a decreased level of DHT was noted [11]. This therapeutic effect of lowering DHT was similar to that of 5α-reductase inhibitors. Future clinical trials should conduct more research on the interference with sexual drive and function as a side effect. Aside from this, the mechanism regarding phloretin and its relationship with the DHT level was not designed and discussed in the animal model [11]. Although phloretin seemed to be effective in ameliorating a T-induced BPH animal model based on reversing the inflammatory, apoptotic and oxidative stress microenvironments, lowering DHT without affecting 5α-reductase enzyme, this might hint that there are still undiscovered mechanisms of phloretin affecting the androgen axis. Thus, detailed research regarding this could be carried out in future studies.

Another concern regarding suppressing the androgen hormone was that it might cause symptoms related to the central nervous system (CNS), such as dementia [84,85]. These CNS-related impairments could be observed in the therapies targeting AR, such as the second-generation hormone therapies of enzalutamide and apalutamide. The mechanism was reported as being associated with low serum T and penetration of blood-brain barrier (BBB) [84]. In clinical practice, cognitive impairment would most likely occur within 6 months after prescription, and the odds remained high for the next 6 months [85]. Since the suppression of T was necessary in treating metastatic or castration-resistant PCa, lowering the penetration across the BBB would be the only solution of phloretin to exert its treatment effect without impairing cognitive function in PCa patients. As a matter of fact, low accumulation in the brain did make darolutamide possess less CNS-related side effects than that of enzalutamide and apalutamide [84]. Thus, the phloretin concentration in the brain could be another determinant parameter of it in treating PCa without causing cognitive impairment. Thus, studies would be worth conducting.

Judging from past clinical experiences, Cox inhibitors remained the primary concern in application in clinical practice [86,87,88]. Cox-1 and Cox-2 displayed differences in affecting gastrointestinal and cardiovascular events. Cox-1 related to platelet aggregation, thromboxane A2, endothelial function, and prostaglandins I_2_. Inhibition of Cox-1 would be more likely to be associated with cardiovascular events. In PCa and BPH, the role of Cox-2 was more prominent [89], and phloretin also exerted its treatment effect via inhibiting Cox-2 [11,12,13,14,15]. Regarding Cox-2, it is essential in gastrointestinal endothelial protection. Inhibition of Cox-2 might be associated with gastrointestinal risks, such as ulcers [86]. Thus, the clear cardiovascular or gastrointestinal risks of phloretin need to be clarified in future clinical trials, and further human clinical trials regarding its safety and effective dosages are worth conducting.

Generally, phloretin currently seems to be promising in inhibiting both PCa and BPH via its anti-inflammatory and pro-apoptotic abilities. Although the roles of ROS in BPH and PCa were distinct, this could all be modulated by phloretin. However, preclinical studies regarding this are still limited, including safety, potential side effects, and unexplained mechanisms. These limitations should be clarified prior to the uses of BPH since there has been only one study published so far [11]. In summary, aside from the uncertainties from available studies thus far, phloretin could be a potential remedy in treating BPH, metastatic PCa, castration-sensitive PCa, and castration-insensitive PCa, and the respective effective dosages are worth being explored.

## Figures and Tables

**Table 1:** General facts about phloretin.

Origins	Leaves of apple trees
Category	Flavonoid; dihydrochalcones
Chemical structure	3-(4-Hydroxyphenyl)-1-(2,4,6-trihydroxyphenyl)-1-propanone
Antioxidant activity	BDE (tested with B3LYP/6-311 + G(d,p) basis set in water): 79.36 kcal/mol The least value of IPs (tested with B3LYP/6-311 + G(d,p) basis set in water): 139.48 kcal/mol
General biological activities in the published literature	Anti-inflammatory ability; anti-oxidative ability; pro-apoptotic ability; anti-proliferative ability

**Table 2:** Treatment effects on cellular characteristics and different biomarkers of 100-μM phloretin among different cell lines of PCa. (↓: decreased, ↑: increased).

	PC3	DU145	LNCaP
Cells’ viability (in MTT assay)	Decreased ↓ [13]	Decreased ↓ [13]	Decreased ↓ [13]
Cells’ viability (in CCK8 assay)	Decreased ↓ [13]	Decreased ↓ [13]	Decreased ↓ [13]
Cells’ proliferation (in Hoechst assay)	Decreased ↓ [15]	-	Decreased ↓ [15]
Biomarkers for cells’ proliferation	VEGF ↓ [13]; CCND1 ↓ [13]; CCNB1 ↓ [13]	-	VEGF ↓ [13]; CCND1 ↓ [13]; CCNB1 ↓ [13];
Reactive oxygen species	Increased [12]	Increased [12]	-
Oxidative stress	Increased [12]	Increased [12]	-
Biomarkers of antioxidant enzymes	Catalase ↓ [12]; SOD2 ↓ [12]; GPx1 ↓ [12]; GPx3 ↓ [12]	Catalase ↓ [12]; SOD2 ↓ [12]; GPx1 ↓ [12]; GPx3 ↓ [12]	-
Biomarkers of wnt/β-catenin signaling pathway	β-catenin ↓ [12]; TCF4 ↓ [12]; FoxA2 ↓ [12]; c-Myc ↓ [12]	β-catenin ↓ [12]; TCF4 ↓ [12]; FoxA2 ↓ [12]; c-Myc ↓ [12]	-
Biomarkers of extrinsic apoptosis	Caspase3/8 ↑ [13]; PARP-1 ↑ [13]	-	TRAIL ↑ [14]; Caspase3/8 ↑ [13]; PARP-1 ↑ [13]
Biomarkers of intrinsic apoptosis	Caspase3/9 ↑ [13]; Bax ↑ [13]; Bcl2 ↓ [13]; XIAP ↓ [13]; Survivin ↓ [13];	-	Caspase3/9 ↑ [13]; Bax ↑ [13]; Bcl2 ↓ [13]; XIAP ↓ [13]; Survivin ↓ [13];

## Data Availability

Not applicable.
